# High executive functioning is associated with reduced posttraumatic stress after trauma exposure among male U.S. military personnel

**DOI:** 10.3389/fpsyg.2023.1181055

**Published:** 2023-09-25

**Authors:** Sabrina R. Liu, Tyler M. Moore, Ruben C. Gur, Caroline Nievergelt, Dewleen G. Baker, Victoria Risbrough, Dean T. Acheson

**Affiliations:** ^1^Department of Human Development, California State University San Marcos, San Marcos, CA, United States; ^2^Department of Psychiatry, University of Pennsylvania, Philadelphia, PA, United States; ^3^Center of Excellence for Stress and Mental Health, VA San Diego Healthcare System, La Jolla, CA, United States; ^4^Department of Psychiatry, University of California, San Diego, La Jolla, CA, United States

**Keywords:** executive function, PTSD, posttraumatic stress, trauma, military, deployment

## Abstract

**Introduction:**

Evidence suggests that executive function (EF) may play a key role in development of PTSD, possibly influenced by factors such as trauma type and timing. Since EF can be improved through intervention, it may be an important target for promoting resilience to trauma exposure. However, more research is needed to understand the relation between trauma exposure, EF, and PTSD. The goal of this study was to improve understanding of EF as a potential antecedent or protective factor for the development of PTSD among military personnel.

**Method:**

In a cohort of U.S. Marines and Navy personnel (*N* = 1,373), the current study tested the association between exposure to traumatic events (pre-deployment and during deployment) and PTSD severity, and whether EF moderated these associations. Three types of pre-deployment trauma exposure were examined: *cumulative exposure*, which included total number of events participants endorsed as having happened to them, witnessed, or learned about; *direct exposure*, which included total number of events participants endorsed as having happened to them; and *interpersonal exposure*, which included total number of interpersonally traumatic events participants’ endorsed. EF was measured using the Penn Computerized Neurocognitive Battery.

**Results:**

EF was associated with less PTSD symptom severity at pre-deployment, even when adjusting for trauma exposure, alcohol use, traumatic brain injury, and number of years in the military. EF also moderated the relation between cumulative trauma exposure and interpersonal trauma exposure and PTSD, with higher EF linked to a 20 and 33% reduction in expected point increase in PTSD symptoms with cumulative and interpersonal trauma exposure, respectively. Finally, higher pre-deployment EF was associated with reduced PTSD symptom severity at post-deployment, independent of deployment-related trauma exposure and adjusting for pre-deployment PTSD.

**Conclusion:**

Our results suggest that EF plays a significant, if small role in the development of PTSD symptoms after trauma exposure among military personnel. These findings provide important considerations for future research and intervention and prevention, specifically, incorporating a focus on improving EF in PTSD treatment.

## Introduction

Prior research suggests that executive function (EF), a group of mental processes (inhibition, working memory, and cognitive flexibility) necessary for concentration and attention, may be an important neurocognitive factor in both risk for, and development of, Posttraumatic Stress Disorder (PTSD). Individuals with PTSD experience deficits in multiple components of EF including working memory, sustaining vigilance and attention, and response inhibition (Vasterling et al., 2009; Martínez et al., 2016; Jacob et al., 2019; Scott et al., 2020). One extant hypothesis is that deficits in EF may represent a neurocognitive subtype of PTSD, possibly influenced by factors such as trauma type or age of trauma exposure (Scott et al., 2015; Jagger-Rickels et al., 2021). Others suggest that disrupted EF is a vulnerability factor for the development or worsening of PTSD symptom severity (Aupperle et al., 2012; Jacob et al., 2019; Scott et al., 2020). Importantly, there is growing evidence that EF can be targeted and improved through physical activity interventions (Sung et al., 2021), cognitive and social rehabilitation training (Novakovic-Agopian et al., 2018; McCarron et al., 2019; Maruyama et al., 2021), and mindfulness-based interventions (Poissant et al., 2020). Given that EF is a potentially malleable risk factor for the development and/or severity of PTSD symptoms, it may be an important target for improving resilience to trauma exposure. However, more research is needed to understand if and how EF influences the relation between trauma exposure and PTSD symptom severity. Using data from the Marine Resiliency Study, a prospective, longitudinal study of PTSD in Active-Duty Marines and Navy Corpsmen, we tested the hypothesis that EF modulates PTSD risk and symptom development after trauma exposure.

In addition to current uncertainty about the role of EF in risk for, and development of PTSD, questions remain regarding the relative impact of different forms of trauma exposure on PTSD symptoms (Kessler et al., 2017; Guina et al., 2018). One large, multi-country survey study found that risk for PTSD increased after trauma exposure, but that the relative risk varied significantly by trauma type, with interpersonally violent traumas carrying the highest level of risk (Kessler et al., 2017). Another study of mental health outpatients found that combat and sexual trauma were associated with greatest PTSD severity, compared to other types such as physical assault or witnessing violence (Guina et al., 2018). These results mirror results of Jakob et al. in a sample of Veterans (Jakob et al., 2017). Similarly, Sheriff and colleagues found that experience of any interpersonal trauma (such as sexual assault or stalking) was significantly linked to meeting the diagnostic criteria for PTSD within the past year, in a sample of military employed as well as in a sample of civilian men. The association between the experience of any non-interpersonal trauma (such as car accidents or major natural disasters) and PTSD was not statistically significant in either cohort (Sheriff et al., 2019). In contrast, Reger et al. (2019) reported that interpersonally traumatic events did not explain mental health outcomes (depression, anxiety, and PTSD symptoms) above and beyond non-interpersonally traumatic events in their sample of active-duty soldiers.

With the goal of improving understanding of EF as a potential antecedent or protective factor for the development of PTSD among military personnel, the current study tested the hypothesis that higher pre-deployment EF promoted better mental health outcomes among a cohort of U.S. Marines. We tested this hypothesis in two ways. First, we used a cross-sectional design to test whether higher EF was directly associated with less severe PTSD symptoms (a main/promotive effect) at the pre-deployment time point. We then tested whether EF moderated (i.e., mitigated) the association between different types of trauma exposure (cumulative lifetime trauma exposure, lifetime direct trauma exposure, and lifetime interpersonal trauma exposure) and PTSD symptom severity at pre-deployment (an interaction/protective effect). Second, we used a longitudinal design to test the hypothesis that greater pre-deployment EF would be associated with fewer PTSD symptoms at post deployment, and that EF would moderate (buffer) the relation between more intense combat experience and greater PTSD symptoms post-deployment.

## Method

### Participants

Study participants were a cohort of 1,373 U.S. male Marines enrolled in a prospective longitudinal study beginning in 2008, of factors related to risk and resilience for deployment-related stress. Assessments were collected at two timepoints-approximately 1 week prior to deployment, and 4–6 months following return from deployment. Of participants who completed the pre-deployment visit, 38% were lost to follow up. Participants lost to follow up had significantly higher levels of posttraumatic stress symptoms on average at their pre-deployment visit (*M* = 14.57, *SD* = 14.69) compared to those participants who were retained (*M* = 12.67, *SD* = 13.47): *t*(1036.45) = 2.41, *p* = 0.02; therefore, all models testing the prospective hypotheses adjusted for pre-deployment posttraumatic stress symptoms. On average, participants were 22 years old (*SD* = 2.89, *Range* = 18–43, *Median* = 21) and had been in the military for 2.33 years (*SD* = 2.27) at the time of first assessment. Most participants (74%) had completed high school, and most (88%) were white. Participant characteristics are reported in more detail in Table 1.

**Table 1:** Sample characteristics (*N* = 1,373).

	Mean (SD) or %
Male gender	100%
Age	22.16 (2.89)
Years in military	2.33 (2.27)
Previously deployed	60.5%
Race	
Black or African American	3.9%
American Indian or Alaskan Native	1.8%
Asian	2.0%
Native Hawaiian or Pacific Islander	1.1%
White	87.5%
Mixed race	3.6%
Ethnicity	
Hispanic/Latino	25.6%
Education level	
High school or less	74.3%
Beyond high school	25.7%
Rank	
Junior enlisted	73.5%
Non-commissioned officer	25.3%
Commissioned officer	1.2%

### Procedure

All study procedures were approved by the Institutional Review Boards of the Veteran’s Administration San Diego Healthcare System, the University of California San Diego, and the Naval Health Research Center. The current study sample included all participants who fully completed the pre-deployment study visit.

### Measures

#### Predictors

Pre-deployment EF was assessed with the Penn Web-Based Computerized Neurocognitive Battery (Penn WebCNB; Moore et al., 2015), a battery of 13 brief neurocognitive tests designed to assess the neurocognitive domains of executive function, episodic memory, complex cognition, and social cognition. Previous research has assessed the factor structure of the WebCNB in a large community sample of young people ages 8–21 (Moore et al., 2015), however, this battery has not been widely used with male marines and its psychometric properties have not been formally established with this unique population. Therefore, a principal components analysis (PCA) with Promax rotation was employed on all test scores to confirm its factor structure. Based on item loadings, conceptual judgement of the research team, and provision of interpretable factor structures, a three-component solution emerged. The three components aligned with executive function, complex reasoning, and memory domains. Specifically, the EF component was made up of performance on tests assessing the core executive function processes of working memory and attention (i.e., the Continuous Performance Test, Short Letter-N-Back Test, and GO-NO-GO test). Participants’ EF component scores were utilized in the current study to represent their level of EF. Full details on the PCA and Penn WebCNB subtests are included in the Supplemental material.

Pre-deployment Trauma Exposure was assessed with the Life Events Checklist (LEC; Gray et al., 2004), which asks participants whether they have experienced any of 17 different potentially traumatic experiences, such as a natural disaster, combat or exposure to a war zone, or sexual or physical assault. For each event, participants indicate if it happened to them personally, they witnessed it happen to someone else, they learned about it happening to someone close to them, or if they are not sure or it does not apply. Similar to other studies of military personnel (Reger et al., 2019), three scores were calculated: *cumulative exposure*, which included total number of events participants endorsed as having happened to them, witnessed, or learned about; *direct exposure*, which included total number of events participants endorsed as having happened to them; and *interpersonal exposure*, which included total number of interpersonally traumatic events that participants endorsed as having happened to them (physical assault, assault with a weapon other than in combat, sexual assault, unwanted or uncomfortable sexual experience, and captivity; Letica-Crepulja et al., 2020; Reich et al., 2021; Berenz et al., 2023). The LEC demonstrates adequate psychometric properties in military populations (Gray et al., 2004).

Exposure to traumatic experiences during the study index deployment was assessed following return from deployment with the Deployment Risk and Resilience Inventory-2 (DRRI-2; Vogt et al., 2013), a collection of 17 scales assessing deployment-related risk and resilience factors. The current study took the average of participant’s scores on two scales— Combat Experiences and Post-battle Experiences— which ask about experiences of combat-related consequences such as being fired on or witnessing an attack, as well as experiencing consequences of combat; for example, seeing dead bodies or taking care of injured people. Responses are rated on a Likert scale from 1 (never) to 6 (daily or almost daily) and summed for a total score. The DRRI demonstrates strong psychometric properties (Vogt et al., 2013).

#### Outcome

Posttraumatic stress symptoms were assessed with the Clinician-Administered PTSD Scale for DSM-4 (CAPS-4), a structured interview assessing symptoms corresponding with DSM-4 criteria for posttraumatic stress disorder. Each symptom is rated for frequency (0 = none of the time to 4 = most or all of the time) and intensity (0 = none to 4 = extreme), and these scores are summed for the overall severity rating used in the current study. The CAPS has demonstrated good psychometric properties (Weathers et al., 2001).

#### Covariates

Risky or hazardous consumption of alcohol was measured with the Alcohol Use Disorders Identification Test (AUDIT; Saunders et al., 1993), a 10-item self-report questionnaire composed of items assessing alcohol intake, alcohol dependence, and adverse consequences of alcohol use in the past 12 months, on a Likert scale from 0 (never) to 4 (daily or almost daily). All items are then summed for a total score. The AUDIT has demonstrated good psychometric properties (Reinert and Allen, 2002) in other studies. In the current sample, internal consistency (assessed with Cronbach’s alpha) was 0.82 and 0.81 at the pre- and post-deployment visits, respectively.

Experience of traumatic brain injury was assessed pre and post deployment with a set of interview questions assessing history of head injuries. Any head injury resulting in self-reported loss of consciousness or altered mental state (e.g., being dazed or confused, seeing stars, or experiencing amnesia immediately afterwards) were classified as TBI. This interview has been used previously in studies of TBI and posttraumatic stress in military samples (Yurgil et al., 2014).

Age, number of years in the military, military rank, and education level were collected via a brief demographic questionnaire (see Baker et al., 2012).

### Data analyses

Descriptive analyses included investigation of data distributions and bivariate correlations. Hierarchical linear regression was utilized to assess whether trauma exposure, EF, and trauma exposure X EF predicted posttraumatic stress symptom severity. Three models were run to assess the impact of different forms of pre-deployment trauma exposure – life events cumulative exposure, life events direct exposure, and life events interpersonal exposure— on pre-deployment posttraumatic stress symptom severity. A fourth model examined the association of deployment-related trauma exposure with post-deployment posttraumatic stress symptom severity. To select model covariates, we examined demographic variables with prior theoretical or empirical support for association with posttraumatic stress symptoms with bivariate correlations, including age, years in military, military rank, education, alcohol use, and traumatic brain injury (Xue et al., 2015; Armenta et al., 2018; Smith and Cottler, 2018; Vasterling et al., 2018). All variables significantly associated (*p* < 0.05) with posttraumatic stress symptoms were included in corresponding models. Hayes’s (2018) PROCESS macro for SPSS was used to plot and probe significant interactions at the intersection of low (16^th^ percentile) and high (84^th^ percentile) values of trauma exposure (Hayes, 2018), and at low, moderate, and high levels of EF (16^th^, 50^th^, and 84^th^ percentile, respectively). In order to probe the specificity of EF associations compared to other cognitive domains, follow-up exploratory analyses were conducted mimicking the above strategy but evaluating Memory and Complex Reasoning separately as the predictor of interest. To explore the utility of using the WebCNB to measure EF for future research and clinical practice, the same regression models were run with a mean composite score of the three tests making up the EF PCA component.

## Results

Table 2 displays means and bivariate associations of study variables. EF was negatively associated with direct and interpersonal life events exposure (*p* < 0.05), as well as pre- and post-deployment CAPS scores (*p* < 0.05). Life events cumulative, direct, and interpersonal exposure were all significantly positively correlated with both pre- and post-deployment CAPS scores (*p* < 0.01). Total combat experience exposure was only associated with CAPS score post-deployment (*p* < 0.01), in the positive direction. Number of years in the military, alcohol use, traumatic brain injury, and being a non-commissioned officer (only for time 2 posttraumatic stress) were significantly associated with posttraumatic stress and included as covariates in subsequent models.

**Table 2:** Means and correlations among trauma exposure, EF, posttraumatic stress symptoms, and demographic factors.

	M (*SD*)	1	2	3	4	5	6	7	8	9	10	11	12	13	14	15	16	17
1. Life events cumulative exposure (T1)	6.80 (3.98)	1																
2. Life events direct exposure (T1)	3.03 (2.13)	0.667^**^	1															
3. Life events interpersonal exposure (T1)	0.85 (0.87)	0.499^**^	0.714^**^	1														
4. Deployment-related trauma exposure (T2)	10.04 (5.84)	0.157^**^	0.166^**^	0.159^**^	1													
5. EF (T1)	0.00 (1)	−0.051	−0.060^*^	−0.068^*^	0.02	1												
6. Posttraumatic stress (T1)	13.40 (13.97)	0.383^**^	0.370^**^	0.309^**^	0.064	−0.085^**^	1											
7. Posttraumatic stress (T2)	15.94 (16.48)	0.224^**^	0.241^**^	0.237^**^	0.266^**^	−0.071^*^	0.458^**^	1										
8. Age	22.16 (2.89)	0.140^**^	0.210^**^	0.034	0.024	0.125^**^	0.016	0.015	1									
9. Years in military	2.33 (2.27)	0.197^**^	0.274^**^	0.074^**^	0.057	0.068^*^	0.112^**^	0.096^**^	0.791^**^	1								
10. Education (% completing beyond high school)	25.7%	0.068^*^	0.067^*^	0.01	0.062	0.144^**^	−0.007	0.004	0.377^**^	0.119^**^	1							
11. Alcohol use (T1)	7.28 (5.64)	0.184^**^	0.142^**^	0.172^**^	0.160^**^	−0.056^*^	0.195^**^	0.180^**^	−0.043	−0.019	−0.006	1						
12. Alcohol use (T2)	6.65 (5.23)	0.139^**^	0.090^**^	0.125^**^	0.162^**^	−0.05	0.138^**^	0.271^**^	−0.079^*^	−0.035	−0.011	0.551^**^	1					
13. Traumatic brain injury (T1)	0.58 (0.49)	0.174^**^	0.197^**^	0.191^**^	0.135^**^	−0.007	0.178^**^	0.122^**^	0.024	0.008	0.027	0.084^**^	0.075^*^	1				
14. Traumatic brain injury (T2)	0.20 (0.40)	0.075^*^	0.076^*^	0.098^**^	0.289^**^	−0.045	0.056	0.234^**^	−0.057	−0.053	−0.014	0.087^**^	0.136^**^	0.100^**^	1			
15. Junior enlisted	0.74 (0.44)	−0.097^**^	−0.135^**^	−0.016	−0.043	−0.095^**^	−0.015	−0.053	−0.541^**^	−0.586^**^	−0.149^**^	0.061^*^	0.096^**^	0.041	0.058	1		
16. Non-commissioned officer	0.25 (0.44)	0.096^**^	0.143^**^	0.023	0.042	0.086^**^	0.026	0.063^*^	0.496^**^	0.576^**^	0.105^**^	−0.057^*^	−0.099^**^	−0.04	−0.061^*^	−0.970^**^	1	
17. Commissioned officer	0.01 (0.11)	0.013	−0.024	−0.029	0.006	0.042	−0.043	−0.039	0.216^**^	0.077^**^	0.185^**^	−0.019	0.006	−0.01	0.01	−0.181^**^	−0.063^*^	1

* *p* ≤ 0.05;

** *p* ≤ 0.01.

### EF and lifetime trauma exposure associations with pre-deployment posttraumatic stress symptoms

**Figure 1:**
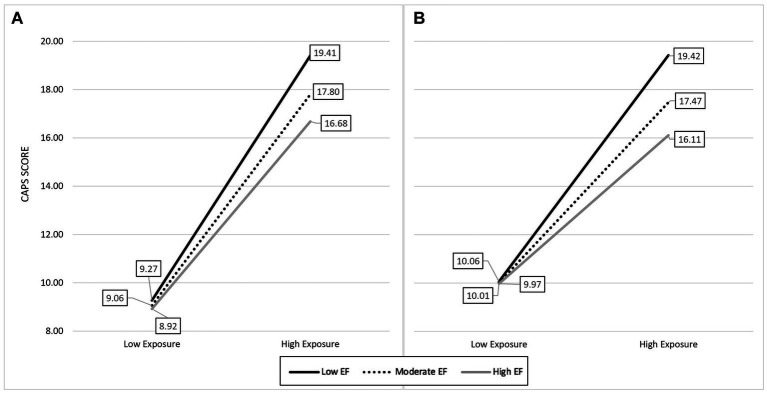
EF buffers the impact lifetime trauma exposure on pre-development posttraumatic stress (**A**: life events cumulative exposure, **B**: life events interpersonal exposure). Low, moderate, and high represent the 16^th^, 50^th^, and 84^th^ percentiles of the predictor and moderate variables.

Results of cross-sectional stepwise regression analyses examining the association of EF, life events exposure, and exposure X EF with pre-deployment posttraumatic stress symptom severity are presented in Tables 3–5. EF significantly predicted posttraumatic stress symptom severity as measured by the CAPS (*p* < 0.05), as did all three forms of exposure (cumulative, direct, and interpersonal, examined separately; all *p’s* < 0.001). These significant relations remained when adjusted for alcohol use, traumatic brain injury, and number of years in the military. Further, EF significantly moderated the relation between cumulative and interpersonal exposure and posttraumatic stress symptom severity (*p* < 0.05, see Figure 1). This interaction effect did not reach significance in the model with direct life events exposure (*p* = 0.14), though was in the same direction as the other effects.

**Table 3:** Main and interaction effects of EF and life events cumulative exposure on pre-deployment posttraumatic stress.

	Estimate	*SE*	Standardized estimate	*p*
Step 1 [*F*(2, 1,348) = 117.85, *p* = 0.00, *R^2^* = 0.15]				
Constant	13.38	0.35		0.000
Exposure	1.32	0.09	0.38	0.000
EF	−0.94	0.35	−0.07	0.008
Step 2 [*F*(3, 1,347) = 80.62, *p* = 0.00, *R^2^* = 0.14]				
Constant	13.34	0.35		0.000
Exposure	1.32	0.09	0.38	0.000
EF	−0.87	0.35	−0.06	0.014
Exposure X EF	−0.20	0.09	−0.06	0.020
Step 3 [*F*(6,1,344) = 50.47, *p* = 0.00, *R^2^* = 0.18]				
Constant	8.44	0.79		0.000
Exposure	1.12	0.09	0.32	0.000
EF	−0.83	0.35	−0.06	0.018
Exposure X EF	−0.17	0.09	−0.05	0.054
Alcohol use	0.29	0.06	0.12	0.000
Traumatic brain injury	3.46	0.71	0.12	0.000
Years in military	0.38	0.16	0.06	0.015

**Table 4:** Main and interaction effects of EF and life events direct exposure on pre-deployment posttraumatic stress.

	Estimate	*SE*	Standardized estimate	*p*
Step 1 [*F*(2, 1,348) = 109.83, *p* = 0.00, *R^2^* = 0.14]				
Constant	13.38	0.35		0.000
Exposure	2.38	0.17	0.37	0.000
EF	−0.90	0.36	−0.06	0.012
Step 2 [*F*(3, 1,347) = 74.27, *p* = 0.00, *R^2^* = 0.14]				
Constant	13.34	0.35		0.000
Exposure	2.37	0.17	0.36	0.000
EF	−0.85	0.36	−0.06	0.017
Exposure X EF	−0.27	0.16	−0.04	0.091
Step 3 [*F*(6,1,344) = 47.83, *p* = 0.00, *R^2^* = 0.18]				
Constant	8.49	0.80		0.000
Exposure	2.02	0.17	0.31	0.000
EF	−0.78	0.35	−0.06	0.027
Exposure X EF	−0.23	0.16	−0.04	0.144
Alcohol use	0.33	0.06	0.14	0.000
Traumatic brain injury	3.41	0.71	0.12	0.000
Years in military	0.24	0.16	0.04	0.138

**Table 5:** Main and interaction effects EF and life events interpersonal exposure on pre-deployment posttraumatic stress.

	Estimate	*SE*	Standardized estimate	*p*
Step 1 [*F*(2, 1,348) = 74.16 *p* = 0.00, *R^2^* = 0.10]				
Constant	13.36	0.36		0.000
Exposure	4.87	0.42	0.30	0.000
EF	−0.91	0.36	−0.07	0.012
Step 2 [*F*(3, 1,347) = 51.43 *p* = 0.00, *R^2^* = 0.10]				
Constant	13.30	0.36		0.000
Exposure	4.84	0.42	0.30	0.000
EF	−0.83	0.37	−0.06	0.024
Exposure X EF	−0.94	0.40	−0.06	0.019
Step 3 [*F*(6,1,344) = 39.12, *p* = 0.00, *R^2^* = 0.15]				
Constant	7.30	0.79		0.000
Exposure	3.88	0.42	0.24	0.000
EF	−0.82	0.36	−0.06	0.022
Exposure X EF	−0.90	0.39	−0.06	0.022
Alcohol use	0.34	0.06	0.14	0.000
Traumatic brain injury	3.66	0.73	0.13	0.000
Years in military	0.65	0.16	0.11	0.000

As can be seen in Figure 1, higher levels of EF moderated the association between cumulative life events exposure and posttraumatic stress symptom severity. Specifically, participants with high EF have approximately a 20% reduction in expected point increase from low to high exposure, compared to those participants with low EF (8 vs. 10 pts). For interpersonal life events exposure, participants with high EF have approximately a 33% reduction in expected point increase compared to participants with low EF (6 vs. 9 pts).

### Pre-deployment EF and deployment-related trauma exposure predicting post-deployment posttraumatic stress symptoms

**Figure 2:**
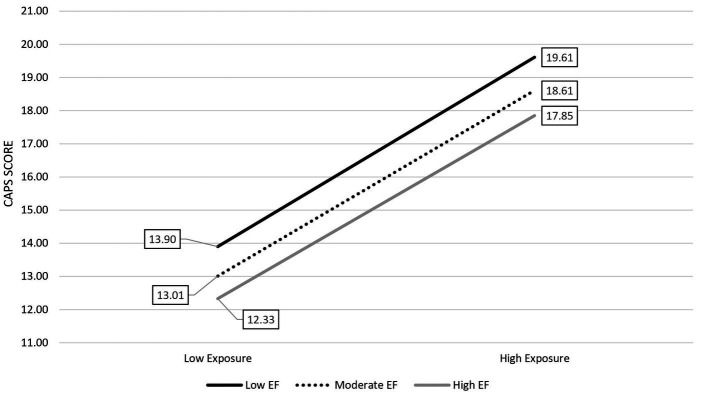
EF is associated with lower levels of posttraumatic stress regardless of combat exposure intensity. Low, moderate, and high represent the 16^th^, 50^th^, and 84^th^ percentiles of the predictor and moderate variables.

Table 6 shows results of linear regression analyses examining the association of pre-deployment executive function, combat experience exposure, and combat experience exposure X pre-deployment EF on post-deployment posttraumatic stress symptom severity, while adjusting for covariates. EF and exposure both had a main effect on posttraumatic stress symptom severity (*p* < 0.001, *p* < 0.05), such that higher levels of pre-deployment EF were associated with less post-deployment posttraumatic stress symptom severity (see Figure 2). Greater combat experience exposure was linked to greater post-deployment posttraumatic stress symptom severity. The interaction of exposure and EF was not significant.

**Table 6:** Main and interaction effects of EF and combat experience exposure on post-deployment posttraumatic stress.

	Estimate	*SE*	Standardized estimate	*p*
Step 1 [*F*(2, 836) = 37.22, *p* = 0.00, *R^2^* = 0.08]				
Constant	16.01	0.55		0.000
Exposure	0.77	0.09	0.27	0.000
EF	−1.50	0.54	−0.09	0.006
Step 2 [*F*(3, 835) = 24.80, *p* = 0.00, *R^2^* = 0.08]				
Constant	16.00	0.55		0.000
Exposure	0.77	0.09	0.27	0.000
EF	−1.50	0.54	−0.09	0.006
Exposure X EF	−0.02	0.09	−0.01	0.812
Step 3 [*F*(9, 829) = 43.06, *p* = 0.00, *R^2^* = 0.32]				
Constant	3.65	1.15		0.001
Exposure	0.47	0.09	0.17	0.000
EF	−0.96	0.47	−0.06	0.043
Exposure X EF	−0.01	0.08	0.00	0.914
Alcohol use	0.56	0.09	0.18	0.000
Traumatic brain injury	6.64	1.25	0.16	0.000
Years in military	0.27	0.29	0.04	0.339
Non-commissioned officer	2.51	1.41	0.06	0.074
Pre-deployment life events cumulative exposure	0.03	0.14	0.01	0.846
Pre-deployment posttraumatic stress	0.48	0.04	0.39	0.000

### Follow-up exploratory analyses

Supplementary Tables S3–S10 show the results of regression analyses with Memory and Complex Reasoning as predictors. For both predictors, no main effects (*ps* > .18) nor interaction (*ps* > .07) effects were significant. Supplementary Tables S11–S15 show the results of regression analyses with the mean composite EF score. Results closely mirror those presented in the main manuscript.

## Discussion

The results of this study suggest that EF plays a significant, if modest role in the development of PTSD symptoms after trauma exposure. Specifically, in a large cohort of U.S. Marines, we found that higher EF was associated with less posttraumatic stress symptom severity both at pre-deployment and post-deployment. These relations were significant even when adjusting for trauma exposure, alcohol use, traumatic brain injury, and number of years in the military. Further, supplemental analyses demonstrate that these findings are unique to EF and do not extend to other aspects of neurocognition, such as complex reasoning and memory (see Supplementary Tables S3–S10). We also found support for our prediction that EF would moderate the link between trauma exposure and PTSD symptoms, with effects being greatest in regard to cumulative lifetime trauma exposure and interpersonal trauma exposure. Specifically, the expected increase in PTSD symptoms for Marines and accompanying Navy personnel with high versus low cumulative trauma exposure was 20% greater for those with low EF, compared to those with high EF. For service members with high versus low interpersonal trauma exposure, higher EF was associated with a 33% reduction in expected point increase compared to those with low EF. The interaction of EF and direct lifetime trauma exposure, while not significant (*p* < 0.14), was in the same direction as the effect of EF X other forms of trauma exposure. Finally, higher pre-deployment EF was associated with reduced posttraumatic stress severity at post-deployment, independent of deployment-related trauma exposure. Taken together, these findings suggest that higher levels of EF are linked to less severe PTSD symptoms, adding to existing literature showing that impaired cognitive functioning, including elements of EF, is a significant and independent risk factor for PTSD in trauma exposed individuals (Olff et al., 2014; Schultebraucks et al., 2021; Mathew et al., 2022).

In our study, when examining the impact of EF on the relation between lifetime trauma exposure and PTSD symptoms, we see evidence that its protective effect is greater for those with higher levels of trauma exposure. Evidence from developmental research suggests that the various components of EF begin to develop in the first few years of life, strengthen significantly throughout childhood and early adolescence, and reach stability by late adolescence (Best and Miller, 2010; Friedman et al., 2016). Given the shared neural circuity of EF and PTSD and importance of developmental timing to EF, it may be that trauma exposure in childhood and adolescence has a strong disruptive impact on developing EF. In turn, lower EF is a vulnerability factor for the development of subsequent PTSD symptoms. In support of this hypothesis, one recent systematic review and meta-analysis found that among children with traumatic experiences with and without PTSS, those with PTSS had significantly lower EF (Nyvold et al., 2022).

When solely examining the protective impact of EF in the context of deployment-related trauma exposure and PTSD symptoms, the magnitude of effect remains constant regardless of severity of trauma exposure. More research is needed to understand these findings, but one hypothesis is that the severity or recency of combat exposure, regardless of amount, is enough that the buffering effect of EF is equivalent across all levels.

The EF measure employed in this study was composed of tasks measuring both working memory (WM) and attention. WM impairments in PTSD are well-documented and have been associated with altered neural function in the fronto-parietal central executive network (Patel et al., 2012). These impairments are thought to reflect a decreased ability to filter threatening information out of memory, while also increasing the storage of task-irrelevant threatening distractors (Stout et al., 2013, 2015). This imbalance in the filtering and retention of threat information may result in an increased occurrence of threat-related cognitions characteristic of re-experiencing symptoms, a core symptom of PTSD. PTSD has also been associated with impaired WM in emotional situations, potentially leading to impaired interpersonal and occupational function (Schweizer and Dalgleish, 2011). Impairments in attentional processes are also well-documented in PTSD (Bar-Haim et al., 2007). Diminished response inhibition ability, which is particularly relevant to the attentional tasks used here, are the CPT and Go-No-Go, as well as difficulties disengaging attention from stimuli perceived as threatening (Aupperle et al., 2012) These difficulties may contribute to PTSD symptoms across a number of domains, such as hyperarousal/vigilance, intrusive memories, and lowered ability to focus on tasks.

There are several limitations for consideration when interpreting our study findings. Although we utilize data from two timepoints (pre- and post-deployment), there is also an important focus on trauma exposure in childhood and adolescence that relies on retrospective report. Future prospective studies examining the impact of trauma on development of PTSD and EF, with more specificity as to exact ages/developmental periods of exposure, could add important supporting evidence to the findings of this study. The racial/ethnic distribution of this participant sample (88% white) differed from that of the entire Marine Corps in 2008 (77% white among E1-E9, 82% white among officers O1–O10; Department of Defense Office of Diversity Management and Equal Opportunity, (2023); future research should examine these constructs in more diverse samples. Last, it is important to note that our findings are correlational in nature, and therefore it is impossible to assert true causality between the main constructs under study (EF, trauma exposure, PTSD symptoms).

Despite this study’s limitations, its results provide important considerations for future research and intervention and prevention. First, results provide evidence as to the feasibility of utilizing the WebCNB, and specifically the Continuous Performance Test, Short Letter-N-Back Test, and GO-NO-GO test, to assess EF among male marines. Results of analyses utilizing the EF component versus the EF composite scores closely aligned, suggesting the utility of a mean composite score approach for future research and clinical practice. Study results also highlight the importance of approaches to PTSD intervention and prevention being developmentally informed. Specifically, intervention for youth and adolescents exposed to trauma may want to incorporate a focus not only on addressing core symptoms of posttraumatic stress but also training to improve EF. For adults, for example Marines and accompanying Navy personnel preparing for deployment, exercises to improve EF prior to deployment may be protective against the later development of PTSD symptoms after trauma exposure. There is a growing body of literature documenting various ways to improve EF, including through physical activity (Sung et al., 2021), cognitive and social rehabilitation training (Novakovic-Agopian et al., 2018; Jak et al., 2019; McCarron et al., 2019; Maruyama et al., 2021; Lindamer et al., 2022), and mindfulness (Poissant et al., 2020).

Importantly, some studies suggest that EF may impact certain symptom clusters of PTSD more than others, although these findings are not consistent. For example, Mathew and colleagues found that performance on visuospatial working memory tasks was a significant predictor only of the re-experiencing PTSD symptom cluster (Mathew et al., 2022), while Olff and colleagues reported that EF performance was significantly linked to numbing and avoidance symptom clusters, and not reexperiencing or hyperarousal (Olff et al., 2014). Overall, literature shows a strong relation between EF and PTSD, but an important question for further research is to disentangle the complex relations between the individual components of both EF and PTSD.

## Data availability statement

The raw data supporting the conclusions of this article will be made available by the authors, without undue reservation.

## Ethics statement

The studies involving humans were approved by the Veteran’s Administration San Diego Healthcare System, the University of California San Diego, and the Naval Health Research Center. The studies were conducted in accordance with the local legislation and institutional requirements. The participants provided their written informed consent to participate in this study.

## Author contributions

TM, RG, CN, DB, and VR participated in study design. SL, TM, CN, and DA conducted the statistical analyses. SL led manuscript preparation and TM, CN, DB, VR, and DA contributed. All authors contributed to the article and approved the submitted version.

## Funding

Support for this work includes NIMH P50MH096889 (Drs Baker and Risbrough), a VA Career Scientist award, VA Merit Award and NIH R01AA026560 (Risbrough), project No. SDR 09-0128 from the Veterans Administration 615 Health Service Research and Development, the US Marine Corps and Navy Bureau of Medicine and Surgery (Drs Baker, Risbrough, Moore and Gur), the Center of Excellence for Stress and Mental Health (Acheson, Nievergelt, Risbrough, Baker). The project described was also supported by the National Center for Research Resources and the National Center for Advancing Translational Sciences, National Institutes of Health, through Grant UL1 625 TR001414.

## Conflict of interest

The authors declare that the research was conducted in the absence of any commercial or financial relationships that could be construed as a potential conflict of interest.

## Publisher’s note

All claims expressed in this article are solely those of the authors and do not necessarily represent those of their affiliated organizations, or those of the publisher, the editors and the reviewers. Any product that may be evaluated in this article, or claim that may be made by its manufacturer, is not guaranteed or endorsed by the publisher.
