# Supplementary material for: High executive functioning is associated with reduced posttraumatic stress after trauma exposure among male U.S. military personnel
Source: Front Psychol. 2023 Sep 25;14:1181055. doi: 10.3389/fpsyg.2023.1181055 (PMC10560729; doi:10.3389/fpsyg.2023.1181055)
Supplement: Supplementary file 1 [file Data_Sheet_1.docx]

Supplementary Material

High Executive Functioning is Associated with Reduced Posttraumatic Stress after Trauma Exposure

Sabrina Liu, Tyler Moore, Ruben Gur, Caroline Nievergelt, Dewleen G. Baker, Victoria Risbrough, Dean Acheson*

*** Correspondence:** Dean Acheson: Dean.Acheson@va.gov

**1 Supplementary Tables**

In line with previous research approaches to handling large sets of neuropsychological variables (Villalobos et al., 2020), we conducted a principal components analysis (PCA) with Promax rotation (based on theoretical and empirical evidence for correlation of factors) on the Penn WebCNB (Moore et al., 2015) subtest efficiency scores. A three-component solution accounting for 56% of the total covariance in items was selected based on item loadings, conceptual judgement of the research team, and provision of interpretable factor structures. Three subtests— the emotion recognition task, motor praxis test, and measured emotion differentiation test— were dropped from analyses, as they did not load strongly onto any component (all loadings < .4) and their conceptual relation to the three emergent components was ambiguous. Remaining subtest loadings aligned with three overarching domains of neurocognitive functioning that we labeled: “Executive Function,” “Memory,” and “Complex Reasoning.” Component loadings are displayed in Table S1. Bolded items indicate those items assigned to each component for purposes of our analyses. Brief descriptions of the cognitive domains assessed by each subtest are provided in Table S2.

References:

Moore, T. M., Reise, S. P., Gur, R. E., Hakonarson, H., & Gur, R. C. (2015). Psychometric Properties of the Penn Computerized Neurocognitive Battery. *Neurophsychology*, *29*(2), 235–246. https://doi.org/10.1037/neu0000093.Psychometric

Villalobos, D., Caperos, J. M., Bilbao, Á., Bivona, U., Formisano, R., & Pacios, J. (2020). Self-awareness moderates the association between executive dysfunction and functional independence after acquired brain injury. *Archives of Clinical Neuropsychology*, *35*(7), 1059–1068. https://doi.org/10.1093/ARCLIN/ACAA048

**Table S1**

*PCA 3-Component Solution Loadings and Inter-Factor Correlations*

|  | EF Component | Memory Component | Complex Reasoning Component |
| --- | --- | --- | --- |
| Continuous Performance Test | .**79** | .06 | -.08 |
| Penn Matrix Reasoning Test | .00 | -.10 | **.78** |
| Short Letter-N-Back Test | **.71** | .00 | .07 |
| GO-NO-GO test | **.86** | -.03 | .01 |
| Penn Conditional Exclusion Task | .10 | .01 | **.66** |
| Penn Face Memory Test | -.05 | **.84** | -.07 |
| Penn Word Memory Test | .07 | **.76** | -.01 |
| Short Visual Object Learning Test | .02 | **.59** | .14 |
| Short Penn's Logical Reasoning Test | -.11 | .16 | **.58** |
| EF Component | 1 |  |  |
| Memory Component | .249** | 1 |  |
| Complex Reasoning Component | .259** | .254** | 1 |

****p ≤ .01.

**Table S2**

*Neurocognitive Domains Assessed by Penn WebCNB Subtests*

| **Component** | **Subtest** | **Neurocognitive Domain** |
| --- | --- | --- |
| Executive function | Continuous Performance Test | Attention |
|  | Short Letter-N-Back Test | Working Memory |
|  | GO-NO-GO test | Attention |
| Memory | Face Memory Test | Face Memory |
|  | Word Memory Test | Word Memory |
|  | Short Visual Object Learning Test | Spatial Memory |
| Complex Reasoning | Matrix Reasoning | Abstraction and Mental Flexibility |
|  | Conditional Exclusion Task | Abstraction and Mental Flexibility |
|  | Logical Reasoning Test | Verbal Ability |

**Table S3**

*Main and Interaction Effects of Memory and Life Events Cumulative Exposure on Posttraumatic Stress*

|  | Estimate | *SE* | Standardized  Estimate | *P* |
| --- | --- | --- | --- | --- |
| Step 1 (*F*(2, 1348) = 114.25, *p* = .00, *R^2^* = .15) |  |  |  |  |
| Constant | 4.28 | .70 |  | .000 |
| Exposure | 1.34 | .09 | .38 | .000 |
| Memory | .35 | .36 | .03 | .326 |
| Step 2 (*F*(3, 1347) = 77.00, *p* = .00, *R^2^* = .15) |  |  |  |  |
| Constant | 4.30 | .70 |  | .000 |
| Exposure | 1.34 | .09 | .38 | .000 |
| Memory | .36 | .36 | .03 | .310 |
| Exposure X Memory | .13 | .09 | .04 | .131 |
| Step 3 (*F*(6,1344) = 49.14, *p* = .00, *R^2^* = .18) |  |  |  |  |
| Constant | .69 | .85 |  | .421 |
| Exposure | 1.13 | .09 | .32 | .000 |
| Memory | .36 | .35 | .03 | .307 |
| Exposure X Memory | .14 | .09 | .04 | .099 |
| Alcohol Use | .31 | .06 | .12 | .000 |
| Traumatic Brain Injury | 3.56 | .71 | .13 | .000 |
| Years in Military | .35 | .16 | .06 | .023 |

**Table S4**

*Main and Interaction Effects of Memory and Life Events Direct Exposure on Posttraumatic Stress*

|  | Estimate | *SE* | Standardized  Estimate | *P* |
| --- | --- | --- | --- | --- |
| Step 1 (*F*(2, 1348) = 106.30, *p* = .00, *R^2^* = .14) |  |  |  |  |
| Constant | 6.07 | .61 |  | .000 |
| Exposure | 2.41 | .17 | .37 | .000 |
| Memory | .18 | .36 | .01 | .607 |
| Step 2 (*F*(3, 1347) = 72.08, *p* = .00, *R^2^* = .14) |  |  |  |  |
| Constant | 6.10 | .61 |  | .000 |
| Exposure | 2.40 | .17 | .37 | .000 |
| Memory | .19 | .36 | .01 | .604 |
| Exposure X Memory | .29 | .16 | .05 | .070 |
| Step 3 (*F*(6,1344) = 47.03, *p* = .00, *R^2^* = .17) |  |  |  |  |
| Constant | 2.28 | .81 |  | .005 |
| Exposure | 2.05 | .17 | .31 | .000 |
| Memory | .19 | .35 | .01 | .597 |
| Exposure X Memory | .29 | .16 | .05 | .073 |
| Alcohol Use | .34 | .06 | .14 | .000 |
| Traumatic Brain Injury | 3.48 | .71 | .12 | .000 |
| Years in Military | .21 | .16 | .04 | .181 |

**Table S5**

*Main and Interaction Effects of Memory and Life Events Interpersonal Exposure on Posttraumatic Stress*

|  | Estimate | *SE* | Standardized  Estimate | *P* |
| --- | --- | --- | --- | --- |
| Step 1 (*F*(2, 1348) = 70.73, *p* = .00, *R^2^* = .10) |  |  |  |  |
| Constant | 9.16 | .51 |  | .000 |
| Exposure | 4.94 | .42 | .31 | .000 |
| Memory | .11 | .37 | .01 | .765 |
| Step 2 (*F*(3, 1347) = 48.06, *p* = .00, *R^2^* = .10) |  |  |  |  |
| Constant | 9.17 | .51 |  | .000 |
| Exposure | 4.90 | .42 | .31 | .000 |
| Memory | .12 | .37 | .01 | .737 |
| Exposure X Memory | .66 | .41 | .04 | .110 |
| Step 3 (*F*(6,1344) = 37.28, *p* = .00, *R^2^* = .14) |  |  |  |  |
| Constant | 3.99 | .79 |  | .000 |
| Exposure | 3.93 | .42 | .25 | .000 |
| Memory | .19 | .36 | .01 | .600 |
| Exposure X Memory | .56 | .40 | .04 | .165 |
| Alcohol Use | .35 | .06 | .14 | .000 |
| Traumatic Brain Injury | 3.70 | .73 | .13 | .000 |
| Years in Military | .62 | .16 | .10 | .000 |

**Table S6**

*Main and Interaction Effects of Memory and Combat Experience Exposure on Post-Deployment Posttraumatic Stress*

|  | Estimate | *SE* | Standardized  Estimate | *P* |
| --- | --- | --- | --- | --- |
| Step 1 (*F*(2, 837) = 33.16 *p* = .00, *R^2^* = .07) |  |  |  |  |
| Constant | 8.27 | 1.10 |  | .000 |
| Exposure | .77 | .09 | .27 | .000 |
| Memory | -.05 | .55 | .00 | .933 |
| Step 2 (*F*(3, 836) = 22.08, *p* = .00, *R^2^* = .07) |  |  |  |  |
| Constant | 8.27 | 1.10 |  | .000 |
| Exposure | .77 | .09 | .27 | .000 |
| Memory | -.04 | .56 | .00 | .938 |
| Exposure X Memory | .00 | .10 | .00 | .987 |
| Step 3 (*F*(8, 831) = 47.30, *p* = .00, *R^2^* = .31) |  |  |  |  |
| Constant | -.94 | 1.27 |  | .458 |
| Exposure | .47 | .09 | .17 | .000 |
| Memory | -.16 | .49 | -.01 | .739 |
| Exposure X Memory | .00 | .09 | .00 | .980 |
| Alcohol Use | .56 | .09 | .18 | .000 |
| Traumatic Brain Injury | 6.62 | 1.25 | .16 | .000 |
| Years in Military | .49 | .23 | .06 | .032 |
| Pre-Deployment Life Events Cumulative Exposure | .04 | .14 | .01 | .799 |
| Pre-Deployment Posttraumatic Stress | .47 | .04 | .39 | .000 |

**Table S7**

*Main and Interaction Effects of Complex Reasoning and Life Events Cumulative Exposure on Posttraumatic Stress*

|  | Estimate | *SE* | Standardized  Estimate | *P* |
| --- | --- | --- | --- | --- |
| Step 1 (*F*(2, 1348) = 114.74, *p* = .00, *R^2^* = .14) |  |  |  |  |
| Constant | 4.26 | .70 |  | .000 |
| Exposure | 1.34 | .09 | .38 | .000 |
| Complex Reasoning | -.47 | .35 | -.03 | .182 |
| Step 2 (*F*(3, 1347) = 76.47, *p* = .00, *R^2^* = .14) |  |  |  |  |
| Constant | 4.27 | .70 |  | .000 |
| Exposure | 1.34 | .09 | .38 | .000 |
| Complex Reasoning | -.48 | .35 | -.03 | .177 |
| Exposure X Complex Reasoning | -.03 | .09 | -.01 | .762 |
| Step 3 (*F*(6,1344) = 48.77, *p* = .00, *R^2^* = .18) |  |  |  |  |
| Constant | .69 | .85 |  | .417 |
| Exposure | 1.14 | .09 | .32 | .000 |
| Complex Reasoning | -.47 | .35 | -.03 | .180 |
| Exposure X Complex Reasoning | -.02 | .09 | -.01 | .855 |
| Alcohol Use | .31 | .06 | .13 | .000 |
| Traumatic Brain Injury | 3.52 | .71 | .13 | .000 |
| Years in Military | .32 | .16 | .05 | .040 |

**Table S8**

*Main and Interaction Effects of Complex Reasoning and Life Events Direct Exposure on Posttraumatic Stress*

|  | Estimate | *SE* | Standardized  Estimate | *P* |
| --- | --- | --- | --- | --- |
| Step 1 (*F*(2, 1348) = 106.43, *p* = .00, *R^2^* = .14) |  |  |  |  |
| Constant | 6.07 | .61 |  | .000 |
| Exposure | 2.41 | .17 | .37 | .000 |
| Complex Reasoning | -.25 | .35 | -.02 | .484 |
| Step 2 (*F*(3, 1347) = 70.99, *p* = .00, *R^2^* = .14) |  |  |  |  |
| Constant | 6.08 | .61 |  | .000 |
| Exposure | 2.41 | .17 | .37 | .000 |
| Complex Reasoning | -.24 | .35 | -.02 | .494 |
| Exposure X Complex Reasoning | .08 | .16 | .01 | .620 |
| Step 3 (*F*(6,1344) = 46.54, *p* = .00, *R^2^* = .17) |  |  |  |  |
| Constant | 2.27 | .81 |  | .005 |
| Exposure | 2.06 | .17 | .32 | .000 |
| Complex Reasoning | -.29 | .35 | -.02 | .401 |
| Exposure X Complex Reasoning | .09 | .16 | .01 | .572 |
| Alcohol Use | .35 | .06 | .14 | .000 |
| Traumatic Brain Injury | 3.45 | .72 | .12 | .000 |
| Years in Military | .20 | .16 | .03 | .222 |

**Table S9**

*Main and Interaction Effects of Complex Reasoning and Life Events Interpersonal Exposure on Posttraumatic Stress*

|  | Estimate | *SE* | Standardized  Estimate | *P* |
| --- | --- | --- | --- | --- |
| Step 1 (*F*(2, 1348) = 71.29 *p* = .00, *R^2^* = .10) |  |  |  |  |
| Constant | 9.14 | .51 |  | .000 |
| Exposure | 4.95 | .42 | .31 | .000 |
| Complex Reasoning | -.38 | .36 | -.03 | .295 |
| Step 2 (*F*(3, 1347) = 47.84 *p* = .00, *R^2^* = .10) |  |  |  |  |
| Constant | 9.16 | .51 |  | .000 |
| Exposure | 4.92 | .42 | .31 | .000 |
| Complex Reasoning | -.36 | .36 | -.03 | .316 |
| Exposure X Complex Reasoning | .42 | .43 | .03 | .334 |
| Step 3 (*F*(6,1344) = 37.18, *p* = .00, *R^2^* = .14) |  |  |  |  |
| Constant | 3.99 | .79 |  | .000 |
| Exposure | 3.95 | .42 | .25 | .000 |
| Memory | -.32 | .35 | -.02 | .361 |
| Exposure X Complex Reasoning | .36 | .42 | .02 | .393 |
| Alcohol Use | .35 | .06 | .14 | .000 |
| Traumatic Brain Injury | 3.71 | .73 | .13 | .000 |
| Years in Military | .60 | .16 | .10 | .000 |

**Table S10**

*Main and Interaction Effects of Memory and Combat Experience Exposure on Post-Deployment Posttraumatic Stress*

|  | Estimate | *SE* | Standardized  Estimate | *P* |
| --- | --- | --- | --- | --- |
| Step 1 (*F*(2, 837) = 33.92, *p* = .00, *R^2^* = .08) |  |  |  |  |
| Constant | 8.26 | 1.10 |  | .000 |
| Exposure | .77 | .09 | .27 | .000 |
| Complex Reasoning | -.64 | .54 | -.04 | .235 |
| Step 2 (*F*(3, 836) = 22.60, *p* = .00, *R^2^* = .08) |  |  |  |  |
| Constant | 8.28 | 1.10 |  | .000 |
| Exposure | .76 | .10 | .27 | .000 |
| Complex Reasoning | -.63 | .54 | -.04 | .244 |
| Exposure X Complex Reasoning | -.01 | .09 | -.01 | .866 |
| Step 3 (*F*(8, 831) = 47.35, *p* = .00, *R^2^* = .31) |  |  |  |  |
| Constant | -.98 | 1.28 |  | .442 |
| Exposure | .47 | .09 | .17 | .000 |
| Complex Reasoning | -.26 | .47 | -.02 | .582 |
| Exposure X Complex Reasoning | .02 | .07 | .01 | .749 |
| Alcohol Use | .56 | .09 | .18 | .000 |
| Traumatic Brain Injury | 6.59 | 1.26 | .16 | .000 |
| Years in Military | .48 | .23 | .06 | .034 |
| Pre-Deployment Life Events Cumulative Exposure | .04 | .14 | .01 | .780 |
| Pre-Deployment Posttraumatic Stress | .47 | .04 | .39 | .000 |

**Table S11**

*Means and Correlations among Trauma Exposure, EF (composite and component scores), Posttraumatic Stress Symptoms, and Demographic Factors*

|  | *M (SD)* | 1 | 2 | 3 | 4 | 5 | 6 | 7 | 8 | 9 | 10 | 11 | 12 | 13 | 14 | 15 | 16 | 17 |
| --- | --- | --- | --- | --- | --- | --- | --- | --- | --- | --- | --- | --- | --- | --- | --- | --- | --- | --- |
| 1. Life Events Cumulative Exposure (T1) | 6.80 (3.98) | 1 |  |  |  |  |  |  |  |  |  |  |  |  |  |  |  |  |
| 1. Life Events Direct Exposure (T1) | 3.03 (2.13) | .667** | 1 |  |  |  |  |  |  |  |  |  |  |  |  |  |  |  |
| 1. Life Events Interpersonal Exposure (T1) | .85 (.87) | .499** | .714** | 1 |  |  |  |  |  |  |  |  |  |  |  |  |  |  |
| 1. Deployment-Related Trauma Exposure (T2) | 10.04 (5.84) | .157** | .166** | .159** | 1 |  |  |  |  |  |  |  |  |  |  |  |  |  |
| 1. EF (T1) | .00 (1) .01 (1.01) | -.051 *-.052* | -.060* *-.063** | -.068* *-.070** | .02 *. 02* | 1 |  |  |  |  |  |  |  |  |  |  |  |  |
| 1. Posttraumatic Stress (T1) | 13.40 (13.97) | .383** | .370** | .309** | .064 | -.085** *-.085*** | 1 |  |  |  |  |  |  |  |  |  |  |  |
| 1. Posttraumatic Stress (T2) | 15.94 (16.48) | .224** | .241** | .237** | .266** | -.071* *-.068** | .458** | 1 |  |  |  |  |  |  |  |  |  |  |
| 1. Age | 22.16 (2.89) | .140** | .210** | .034 | .024 | .125** *.128*** | .016 | .015 | 1 |  |  |  |  |  |  |  |  |  |
| 1. Years in Military | 2.33 (2.27) | .197** | .274** | .074** | .057 | .068* .*072** | .112** | .096** | .791** | 1 |  |  |  |  |  |  |  |  |
| 1. Education (% completing beyond High School) | 25.7% | .068* | .067* | .01 | .062 | .144** .*140*** | -.007 | .004 | .377** | .119** | 1 |  |  |  |  |  |  |  |
| 1. Alcohol Use (T1) | 7.28 (5.64) | .184** | .142** | .172** | .160** | -.056* *-.060** | .195** | .180** | -.043 | -.019 | -.006 | 1 |  |  |  |  |  |  |
| 1. Alcohol Use (T2) | 6.65 (5.23) | .139** | .090** | .125** | .162** | -.05 *-.05* | .138** | .271** | -.079* | -.035 | -.011 | .551** | 1 |  |  |  |  |  |
| 1. Traumatic Brain Injury (T1) | .58 (.49) | .174** | .197** | .191** | .135** | -.007 -*.033* | .178** | .122** | .024 | .008 | .027 | .084** | .075* | 1 |  |  |  |  |
| 1. Traumatic Brain Injury (T2) | .20 (.40) | .075* | .076* | .098** | .289** | -.045 *.033* | .056 | .234** | -.057 | -.053 | -.014 | .087** | .136** | .100** | 1 |  |  |  |
| 1. Junior Enlisted | .74 (.44) | -.097** | -.135** | -.016 | -.043 | -.095** *-.101*** | -.015 | -.053 | -.541** | -.586** | -.149** | .061* | .096** | 0.041 | .058 | 1 |  |  |
| 1. Non-Commissioned Officer | .25 (.44) | .096** | .143** | .023 | .042 | .086** *.091*** | .026 | .063* | .496** | .576** | .105** | -.057* | -.099** | -0.04 | -.061* | -.970** | 1 |  |
| 1. Commissioned Officer | .01 (.11) | .013 | -.024 | -.029 | .006 | .042 *.047* | -.043 | -.039 | .216** | .077** | .185** | -.019 | .006 | -.01 | .01 | -.181** | -.063* | 1 |

**p ≤* .05, ***p* ≤ .01.

*Note.* Results of analyses with EF composite score are in italics.

**Table S12**

*Main and Interaction Effects of EF (composite and component scores) and Life Events Cumulative Exposure on Pre-Deployment Posttraumatic Stress*

|  | Estimate | | *SE* | | Standardized  Estimate | | *P* | |
| --- | --- | --- | --- | --- | --- | --- | --- | --- |
| Step 1 (*F*(2, 1348) = 117.85, *p* = .00, *R^2^* = .15)  *(F(2, 1348) = 117.77, p = .00, R^2^ = .15)* |  |  |  |  |  |  |  |  |
| Constant | 13.38 | *13.38* | .35 | *.35* |  |  | .000 | *.000* |
| Exposure | 1.32 | *1.32* | .09 | *.09* | .38 | *.38* | .000 | *.000* |
| EF | -.94 | *-.86* | .35 | *.33* | -.07 | *-.07* | .008 | *.008* |
| Step 2 (*F*(3, 1347) = 80.62, *p* = .00, *R^2^* = .14)  *(F(3, 1347) = 80.52, p = .00, R^2^ = .15)* |  |  |  |  |  |  |  |  |
| Constant | 13.34 | *13.34* | .35 | *.35* |  |  | .000 | *.000* |
| Exposure | 1.32 | *1.32* | .09 | *.09* | .38 | *.38* | .000 | *.000* |
| EF | -.87 | *-.80* | .35 | *.33* | -.06 | *-.06* | .014 | *.015* |
| Exposure X EF | -.20 | *-.19* | .09 | *.08* | -.06 | *-.06* | .020 | *.022* |
| Step 3 (*F*(6,1344) = 50.47, *p* = .00, *R^2^* = .18)  *(F(6,1344) = 50.41, p = .00, R^2^ = .18)* |  |  |  |  |  |  |  |  |
| Constant | 8.44 | *8.44* | .79 | *.79* |  |  | .000 | *.000* |
| Exposure | 1.12 | *1.12* | .09 | *.09* | .32 | *.32* | .000 | *.000* |
| EF | -.83 | *-.76* | .35 | *.32* | -.06 | *-.06* | .018 | *.018* |
| Exposure X EF | -.17 | *-.15* | .09 | *.08* | -.05 | *-.05* | .054 | *.058* |
| Alcohol Use | .29 | *.29* | .06 | *.06* | .12 | *.12* | .000 | *.000* |
| Traumatic Brain Injury | 3.46 | *3.47* | .71 | *.71* | .12 | *.12* | .000 | *.000* |
| Years in Military | .38 | *.38* | .16 | *.16* | .06 | *.06* | .015 | *.015* |

*Note.* Results of analyses with EF composite score are in italics. **Table S13**

*Main and Interaction Effects of EF (composite and component scores) and Life Events Direct Exposure on Pre-Deployment Posttraumatic Stress*

|  | Estimate | | *SE* | | Standardized  Estimate | | *P* | |
| --- | --- | --- | --- | --- | --- | --- | --- | --- |
| Step 1 (*F*(2, 1348) = 109.83, *p* = .00, *R^2^* = .14)  *(F(2, 1348) = 109.83, p = .00, R^2^ = .14)* |  |  |  |  |  |  |  |  |
| Constant | 13.38 | *13.38* | .35 | *.35* |  |  | .000 | *.000* |
| Exposure | 2.38 | *2.38* | .17 | *.17* | .37 | *.37* | .000 | *.000* |
| EF | -.90 | *-.81* | .36 | *.33* | -.06 | *-.06* | .012 | *.014* |
| Step 2 (*F*(3, 1347) = 74.27, *p* = .00, *R^2^* = .14)  *(F(3, 1347) = 74.27, p = .00, R^2^ = .14)* |  |  |  |  |  |  |  |  |
| Constant | 13.34 | *13.34* | .35 | *.35* |  |  | .000 | *.000* |
| Exposure | 2.37 | *2.37* | .17 | *.17* | .36 | *.36* | .000 | *.000* |
| EF | -.85 | *-.76* | .36 | *.33* | -.06 | *-.06* | .017 | *.020* |
| Exposure X EF | -.27 | *-.25* | .16 | *.15* | -.04 | *-.04* | .091 | *.087* |
| Step 3 (*F*(6,1344) = 47.83, *p* = .00, *R^2^* = .18)  *(F(6,1344) = 47.83, p = .00, R^2^ = .18)* |  |  |  |  |  |  |  |  |
| Constant | 8.49 | *8.49* | .80 | *.80* |  |  | .000 | *.000* |
| Exposure | 2.02 | *2.02* | .17 | *.17* | .31 | *.31* | .000 | *.000* |
| EF | -.78 | *-.69* | .35 | *.32* | -.06 | *-.05* | .027 | *.032* |
| Exposure X EF | -.23 | *-.21* | .16 | *.14* | -.04 | *-.04* | .144 | *.138* |
| Alcohol Use | .33 | *.33* | .06 | *.06* | .14 | *.14* | .000 | *.000* |
| Traumatic Brain Injury | 3.41 | *3.42* | .71 | *.71* | .12 | *.12* | .000 | *.000* |
| Years in Military | .24 | *.24* | .16 | *.16* | .04 | *.04* | .138 | *.135* |

*Note.* Results of analyses with EF composite score are in italics.

**Table S14**

*Main and Interaction Effects EF (composite and component scores) and Life Events Interpersonal Exposure on Pre-Deployment Posttraumatic Stress*

|  | Estimate | | *SE* | | Standardized  Estimate | | *P* | |
| --- | --- | --- | --- | --- | --- | --- | --- | --- |
| Step 1 (*F*(2, 1348) = 74.16 *p* = .00, *R^2^* = .10)  *(F(2, 1348) = 74.11, p = .00, R^2^ = .10)* |  |  |  |  |  |  |  |  |
| Constant | 13.36 | *13.36* | .36 | *.36* |  |  | .000 | *.000* |
| Exposure | 4.87 | *4.87* | .42 | *.42* | .30 | *.30* | .000 | *.000* |
| EF | -.91 | *-.83* | .36 | *.33* | -.07 | *-.07* | .012 | *.013* |
| Step 2 (*F*(3, 1347) = 51.43 *p* = .00, *R^2^* = .10)  *(F(3, 1347) = 51.05, p = .00, R^2^ = .10)* |  |  |  |  |  |  |  |  |
| Constant | 13.30 | *13.31* | .36 | *.36* |  |  | .000 | *.000* |
| Exposure | 4.84 | *4.84* | .42 | *.42* | .30 | *.30* | .000 | *.000* |
| EF | -.83 | *-.76* | .37 | *.34* | -.06 | *-.06* | .024 | *.024* |
| Exposure X EF | -.94 | *-.79* | .40 | *.37* | -.06 | *-.06* | .019 | *.033* |
| Step 3 (*F*(6,1344) = 39.12, *p* = .00, *R^2^* = .15)  *(F(6,1344) = 38.93, p = .00, R^2^ = .15)* |  |  |  |  |  |  |  |  |
| Constant | 7.30 | *7.30* | .79 | *.79* |  |  | .000 | *.000* |
| Exposure | 3.88 | *3.88* | .42 | *.42* | .24 | *.24* | .000 | *.000* |
| EF | -.82 | *-.75* | .36 | *.33* | -.06 | *-.06* | .022 | *.022* |
| Exposure X EF | -.90 | *-.75* | .39 | *.36* | -.06 | *-.05* | .022 | *.037* |
| Alcohol Use | .34 | *.34* | .06 | *.06* | .14 | *.14* | .000 | *.000* |
| Traumatic Brain Injury | 3.66 | *3.66* | .73 | *.73* | .13 | *.13* | .000 | *.000* |
| Years in Military | .65 | *.65* | .16 | *.16* | .11 | *.11* | .000 | *.000* |

*Note.* Results of analyses with EF composite score are in italics.

**Table S15**

*Main and Interaction Effects of EF (composite and component scores) and Combat Experience Exposure on Post-Deployment Posttraumatic Stress*

|  | Estimate | | *SE* | | Standardized  Estimate | | *P* | |
| --- | --- | --- | --- | --- | --- | --- | --- | --- |
| Step 1 (*F*(2, 836) = 37.22, *p* = .00, *R^2^* = .08)  *(F(2, 836) = 36.77, p = .00, R^2^ = .08)* |  |  |  |  |  |  |  |  |
| Constant | 16.01 | *16.00* | .55 | *.55* |  |  | .000 | *.000* |
| Exposure | .77 | *.77* | .09 | *.09* | .27 | *.27* | .000 | *.000* |
| EF | -1.50 | *-1.30* | .54 | *.50* | -.09 | *-.09* | .006 | *.009* |
| Step 2 (*F*(3, 835) = 24.80, *p* = .00, *R^2^* = .08)  (*F(3, 835) = 24.48, p = .00, R^2^ = .08)* |  |  |  |  |  |  |  |  |
| Constant | 16.00 | *16.00* | .55 | *.55* |  |  | .000 | *.000* |
| Exposure | .77 | *.77* | .09 | *.09* | .27 | *.27* | .000 | *.000* |
| EF | -1.50 | *-1.30* | .54 | *.50* | -.09 | *-.09* | .006 | *.009* |
| Exposure X EF | -.02 | *-.01* | .09 | *.09* | -.01 | *.00* | .812 | *.953* |
| Step 3 (*F*(9, 829) = 43.06, *p* = .00, *R^2^* = .32)  *(F(9, 829) = 42.98, p = .00, R^2^ = .31)* |  |  |  |  |  |  |  |  |
| Constant | 3.65 | *3.64* | 1.15 | *1.15* |  |  | .001 | *.002* |
| Exposure | .47 | *.47* | .09 | *.09* | .17 | *.17* | .000 | *.000* |
| Executive Function | -.96 | *-.82* | .47 | *.44* | -.06 | *-.06* | .043 | *.059* |
| Exposure X EF | -.01 | *.00* | .08 | *.08* | .00 | *.00* | .914 | *.953* |
| Alcohol Use | .56 | *.57* | .09 | *.09* | .18 | *.18* | .000 | *.000* |
| Traumatic Brain Injury | 6.64 | *6.65* | 1.25 | *1.25* | .16 | *.16* | .000 | *.000* |
| Years in Military | .27 | *.28* | .29 | *.29* | .04 | *.04* | .339 | *.332* |
| Non-Commissioned Officer | 2.51 | *2.50* | 1.41 | *1.41* | .06 | *.06* | .074 | *.076* |
| Pre-Deployment Life Events Cumulative Exposure | .03 | *.03* | .14 | *.14* | .01 | *.01* | .846 | *.850* |
| Pre-Deployment Posttraumatic Stress | .48 | *.48* | .04 | *.04* | .39 | *.39* | .000 | *.000* |

*Note.* Results of analyses with EF composite score are in italics.
